# Supplementary material for: In silico Approach for Validating and Unveiling New Applications for Prognostic Biomarkers of Endometrial Cancer
Source: Cancers (Basel). 2021 Oct 9;13(20):5052. doi: 10.3390/cancers13205052 (PMC8534093; doi:10.3390/cancers13205052)
Supplement: Supplementary file 1 [file cancers-13-05052-s001.zip › cancers-1391148-SI.pdf]

cancers

MDPI

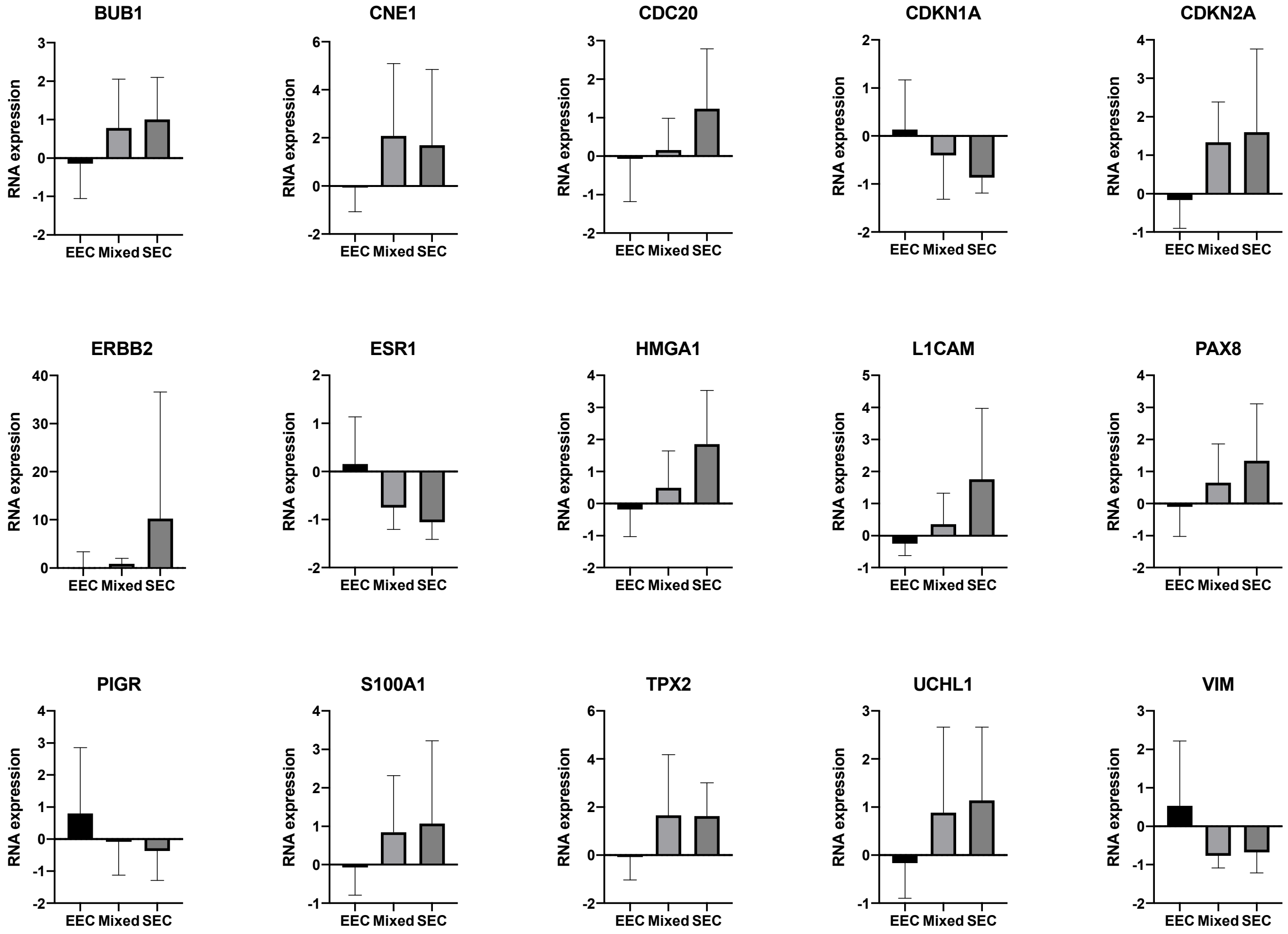

Figure Supplementary 1. Boxplots of the described validated biomarkers for histological type representing different non-endometrioid histologies as different entities (n=271 endometrioid (EEC) type, n=62 non-EEC type including n=10 mixed carcinomas, and n=52 serous (SEC) carcinomas).

A

Gene Name (Protein ID)	Literature	T_RNAseq	C_RNAseq	C_prot
PGR(P06401)	FIGO	IvsII; IvsIV		IvsII
ANXA2(P07355)	FIGO			IvsIV; IIIvsIV
CRP(P02741)	FIGO			IvsIV; IIvsIV; IIIvsIV
ESR1(P03372)	FIGO	IvsIV		
FGA(P02671)	FIGO			IvsIV; IIIvsIV
HMGA1(P17096)	FIGO			IvsIV; IIvsIV; IIIvsIV
LRP1(Q07954)	FIGO			IIIvsIV
PRL(P01236)	FIGO		IIvsIII; IIvsIV	
PTEN(P60484)	FIGO			IvsII
SAA1(P0DJI8)	FIGO			IvsIV; IIIvsIV
TNFAIP8(O95379)	FIGO			IvsIV
AKT1(P31749)				IvsIV; IIIvsIV
CD44(P16070)				IIIvsIV
CDK4(P11802)				IvsIV; IIvsIV
IL8(P10145)				IvsIV; IIvsIV; IIIvsIV
MDK(P21741)				IvsIV; IIvsIV; IIIvsIV
SCGB2A1(O75556)		IvsIV		
SPINT1(O43278)		IvsIV; IIvsIV		
TIMP2(P16035)				IIIvsIV
TRIM27(P14373)				IvsIV; IIvsIV; IIIvsIV
URI(O94763)				IIvsIII
YTHDC1(Q96MU7)				IvsIV; IIIvsIV

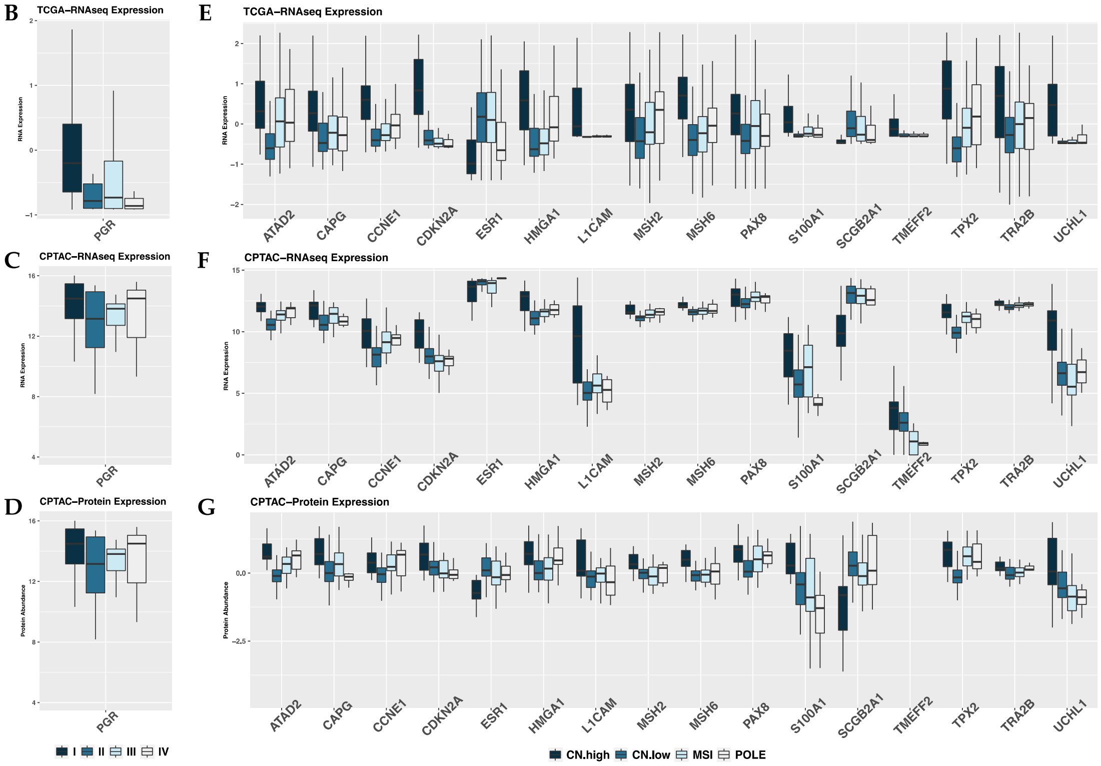

Figure Supplementary 2. Biomarkers related to FIGO stage with its respective boxplots, and molecular classification of the validated biomarkers. (**a**) Table of the proteins that were found differentially expressed between (**a**) Any of the FIGO stages in any of the tested cohorts. Highlighted in yellow, the specific cohort in which that protein was found to be differentially expressed between FIGO stages. Proteins highlighted in blue are those validated in more than one cohort, and therefore, the ones that we considered as validated biomarkers; (**b–d**) Boxplots showing the expression of the significant biomarkers for FIGO stage in each cohort of patients: TCGA RNA-Seq data, CPTAC RNA-Seq data, and CPTAC proteomic data, respectively; (**e–g**) Boxplots showing the expression of the significant biomarkers for molecular classification in each cohort of patients: TCGA RNA-Seq data, CPTAC RNA-Seq data, and CPTAC proteomic data, respectively. *Literature: literature revision from Coll-de la Rubia E et al., 2020* [1]; *T_RNAseq: RNA-Seq data of the TCGA's cohort; C_RNAseq: RNA-Seq data of the CPTAC's cohort; C_prot: proteomic data of the CPTAC's cohort.*

	Function [CC] - Uniprot	EMT - EMTome	Cancer prognostic summary - The Human Protein Atlas	
**Gene Name**	**Function**	**EMT-related proteins**	**Favorable**	**Unfavorable**
**ASRGL1**	Has both L-asparaginase and beta-aspartyl peptidase activity.	No	colorectal, renal	liver
**ATAD2**	Involved in the estrogen-induced cell proliferation and cell cycle progression of breast cancer cells.	No		lung, renal
**BUB1**	Serine/threonine-protein kinase that performs 2 crucial functions during mitosis: it is essential for spindle-assembly checkpoint signaling and for correct chromosome alignment.	No		liver, pancreatic
**CAPG**	Calcium-sensitive protein which reversibly blocks the barbed ends of actin filaments but does not sever preformed actin filaments.	Yes	renal	liver
**CCNE1**	Essential for the control of the cell cycle at the G1/S (start) transition.	No		liver, **ovarian**
**CDC20**	Required for full ubiquitin ligase activity of the anaphase promoting complex/cyclosome (APC/C).	No		liver, pancreatic, renal
**CDKN1A**	Binds to and inhibits cyclin-dependent kinase activity, preventing phosphorylation of critical cyclin-dependent kinase substrates and blocking cell cycle progression.	Yes	renal	lung
**CDKN2A**	Acts as a negative regulator of the proliferation of normal cells by interacting strongly with CDK4 and CDK6.	Yes	head and neck	liver, renal
**ERBB2**	Protein tyrosine kinase that is part of several cell surface receptor complexes, but that apparently needs a coreceptor for ligand binding. In the nucleus is involved in transcriptional regulation. Involved in the transcription of rRNA genes by RNA Pol I and enhances protein synthesis and cell growth.	Yes	renal	pancreatic
**ESR1**	Nuclear hormone receptor. The steroid hormones and their receptors are involved in the regulation of eukaryotic gene expression and affect cellular proliferation and differentiation in target tissues. Also mediates membrane-initiated estrogen signaling involving various kinase cascades.	Yes		
**FASN**	Fatty acid synthetase is a multifunctional enzyme that catalyzes the de novo biosynthesis of long-chain saturated fatty acids starting from acetyl-CoA and malonyl-CoA in the presence of NADPH.	No		**cervical**, renal
**HDGF**	[Isoform 1]: Acts as a transcriptional repressor. Has mitogenic activity for fibroblasts. Heparin-binding protein.	Yes	**ovarian**	liver
**HMGA1**	HMG-I/Y bind preferentially to the minor groove of A+T rich regions in double-stranded DNA.They are also involved in the transcription regulation of genes containing, or in close proximity to A+T-rich regions.	Yes		liver, lung, pancreatic
**L1CAM**	Neural cell adhesion molecule involved in the dynamics of cell adhesion and in the generation of transmembrane signals at tyrosine kinase receptors.	Yes		head and neck, lung, renal
**MACC1**	Acts as a transcription activator for MET and as a key regulator of HGF-MET signaling. Promotes cell motility, proliferation and hepatocyte growth factor (HGF)-dependent.	Yes	renal	
**MCM6**	Acts as component of the MCM2-7 complex (MCM complex) which is the putative replicative helicase essential for 'once per cell cycle' DNA replication initiation and elongation in eukaryotic cells.	No		liver, melanoma, renal
**MCM7**	Acts as component of the MCM2-7 complex (MCM complex) which is the putative replicative helicase essential for 'once per cell cycle' DNA replication initiation and elongation in eukaryotic cells. Required for S-phase checkpoint activation upon UV-induced damage.	Yes	**cervical**	liver
**MSH2**	Component of the post-replicative DNA mismatch repair system (MMR).	No		liver, pancreatic
**MSH6**	Component of the post-replicative DNA mismatch repair system (MMR).	No		liver, renal
**PAX8**	Transcription factor for the thyroid-specific expression of the genes exclusively expressed in the thyroid cell type, maintaining the functional differentiation of such cells.	No		
**PGR**	The steroid hormones and their receptors are involved in the regulation of eukaryotic gene expression and affect cellular proliferation and differentiation in target tissues.	Yes		
**PIGR**	Mediates selective transcytosis of polymeric IgA and IgM across mucosal epithelial cells. Binds polymeric IgA and IgM at the basolateral surface of epithelial cells.	No	**breast**, renal	
**PTK2**	Non-receptor protein-tyrosine kinase that plays an essential role in regulating cell migration, adhesion, spreading, reorganization of the actin cytoskeleton, formation and disassembly of focal adhesions and cell protrusions, cell cycle progression, cell proliferation and apoptosis.	Yes		**breast**
**S100A1**	Small calcium binding protein that plays important roles in several biological processes such as Ca(2+) homeostasis, chondrocyte biology and cardiomyocyte regulation.	No		renal
**SCGB2A1**	May bind androgens and other steroids, may also bind estramustine, a chemotherapeutic agent used for prostate cancer. May be under transcriptional regulation of steroid hormones.	No	colorectal, renal	
**TMEFF2**	May be a survival factor for hippocampal and mesencephalic neurons. The shedded form up-regulates cancer cell proliferation, probably by promoting ERK1/2 phosphorylation.	No	Gene product is not prognostic	
**TPX2**	Spindle assembly factor required for normal assembly of mitotic spindles.	No		liver, lung, pancreatic, renal
**TRA2B**	Sequence-specific RNA-binding protein which participates in the control of pre-mRNA splicing. Can either activate or suppress exon inclusion.	No	**ovarian**	liver
**UCHL1**	Ubiquitin-protein hydrolase involved both in the processing of ubiquitin precursors and of ubiquitinated proteins (Probable). This enzyme is a thiol protease that recognizes and hydrolyzes a peptide bond at the C-terminal glycine of ubiquitin. Also binds to free monoubiquitin and may prevent its degradation in lysosomes (By similarity).	Yes		urothelial
**VIM**	Vimentins are class-III intermediate filaments found in various non-epithelial cells, especially mesenchymal cells. Vimentin is attached to the nucleus, endoplasmic reticulum, and mitochondria, either laterally or terminally.	Yes		renal

Figure Supplementary 3. Description of the functions of the proteins described as validated biomarkers in our study, their relation to the epithelial-mesenchymal transition, and their prognostic behavior in other types of cancer. Highlighted in bold are gynecological cancers in which a prognostic association of that specific protein has been described. Source: Uniprot [2], EMTome [3], and The Human Protein Atlas (www.proteinatlas.org).

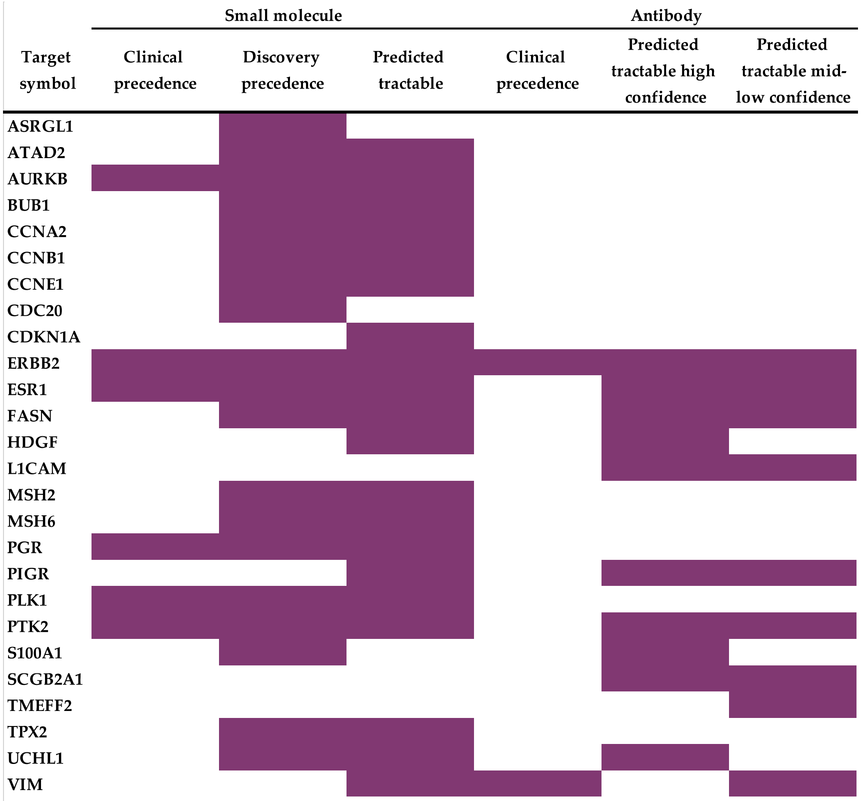

**AURKB**	AT-9283 (multiple myeloma); TOZASERTIB (leukemia); DANUSERTIB (multiple myeloma); ENMD-2076 (soft tissue sarcoma); BI-811283 (acute myeloid leukemia); BARASERTIB (acute myeloid leukemia); CHIAURANIB (ovarian cancer); ILORASERTIB (cancer); TAK-901 (acute myeloid leukemia); SNS-314 (neoplasm); KW-2449 (acute myeloid leukemia); CENISERTIB (lymphoid neoplasm); AMG-900 (cancer); CYC-116 (neoplasm); TTP-607 (lymphoma); GSK-1070916 (neoplasm); MK-6592 (neoplasm); PF-03814735 (neoplasm)
**ERBB2** **(Small molecules)**	AFATINIB (non-small cell lung carcinoma); NERATINIB (neoplasm); VANDETANIB (thyroid cancer); LAPATINIB (neoplasm); DACOMITINIB (non-small cell lung carcinoma); TUCATINIB (HER2 Positive Breast Carcinoma); PYROTINIB (breast cancer); TESEVATINIB (non-small cell lung carcinoma); POZIOTINIB (non-small cell lung carcinoma); VARLITINIB (cholangiocarcinoma); BMS-690514 (non-small cell lung carcinoma); SAPITINIB (breast cancer); CP-724714 (metastasis); CANERTINIB (breast neoplasm); HEMAY-022 (breast cancer); CUDC-101 (head and neck malignant neoplasia); AEE-788 (glioblastoma multiforme); MUBRITINIB (breast neoplasm); AC-480 (cancer); TAK-285 (cancer); MP-412 (neoplasm); JNJ-26483327 (cancer)
**ERBB2** **(Antibodies)**	TRASTUZUMAB EMTANSINE (neoplasm); TRASTUZUMAB (breast carcinoma); ; PERTUZUMAB (neoplasm); TRASTUZUMAB DERUXTECAN (breast cancer); MARGETUXIMAB (breast cancer); ERTUMAXOMAB (breast cancer); GANCOTAMAB (breast cancer); T-DM1 (breast cancer)
**ESR1**	ESTRADIOL (hypogonadism); ESTRADIOL VALERATE (infertility); BAZEDOXIFENE (obesity); CLOMIPHENE (anovulation); FULVESTRANT (breast carcinoma); ETHINYL ESTRADIOL (infertility); TOREMIFENE (breast carcinoma); ESTROGENS, CONJUGATED (postmenopausal osteoporosis); TAMOXIFEN (breast cancer); DIETHYLSTILBESTROL (neoplasm); ESTRIOL (urinary tract infection); POLYESTRADIOL PHOSPHATE (neoplasm); DIETHYLSTILBESTROL DIPHOSPHATE (neoplasm); OSPEMIFENE (sexual dysfunction); LASOFOXIFENE (osteoporosis); ARZOXIFENE (postmenopausal osteoporosis); SYNTHETIC CONJUGATED ESTROGENS, B (menopause); AFIMOXIFENE (breast ductal carcinoma in situ); RAD1901 (Hot flashes); FISPEMIFENE (hypogonadism); BRILANESTRANT (breast cancer); ESTROGENS, ESTERIFIED (breast cancer); SR16234 (breast cancer); ESTRONE (obesity); GTX-758 (prostate cancer); ACOLBIFENE (breast cancer)
**PGR**	PROGESTERONE (infertility); ULIPRISTAL (uterine fibroid); NORETHINDRONE ACETATE (hypogonadism); ULIPRISTAL ACETATE (uterine fibroid); DROSPIRENONE (Dysmenorrhea); DYDROGESTERONE (premature birth); LEVONORGESTREL (Menorrhagia); HYDROXYPROGESTERONE CAPROATE (premature birth); MEDROXYPROGESTERONE ACETATE (hemorrhage); CYPROTERONE ACETATE (acne); DESOGESTREL (uterine fibroid); ETONOGESTREL (HIV infection); MEGESTROL ACETATE (precocious puberty); MIFEPRISTONE (persian gulf syndrome); NORGESTIMATE (acne); NORETHINDRONE (postpartum depression); TELAPRISTONE ACETATE (uterine fibroid); DANAZOL (diabetic macular edema); ASOPRISNIL (leiomyoma); NORELGESTROMIN (Metrorrhagia); NORGESTREL (endometrial cancer); LONAPRISAN (breast cancer); PF-05019702 (uterine fibroid); ONAPRISTONE (neoplasm)
**PLK1**	VOLASERTIB (acute myeloid leukemia); BI-2536 (acute myeloid leukemia); ONVANSERTIB (prostate cancer); TAK-960 (cancer); MK-1496 (neoplasm); GSK-461364 (non-Hodgkins lymphoma); CAFUSERTIB (acute myeloid leukemia)
**PTK2**	DEFACTINIB (pancreatic ductal adenocarcinoma); GSK-2256098 (pancreatic adenocarcinoma); VS-4718 (pancreatic carcinoma); CEP-37440 (neoplasm); BI-853520 (neoplasm)
**VIM**	PRITUMUMAB (glioma)

Figure Supplementary 4. Available drugs against validated proteins divided in small molecules and antibodies. A description of current clinical applications with the commercial names is given below. Source: Open Target Platform [4].

References

1. Coll-de la Rubia, E.; Martinez-Garcia, E.; Dittmar, G.; Gil-Moreno, A.; Cabrera, S.; Colas, E. Prognostic Biomarkers in Endometrial Cancer: A Systematic Review and Meta-Analysis. *J. Clin. Med.* **2020**, *9*, 1900, doi:10.3390/jcm9061900.

2. Consortium, T.U.; Bateman, A.; Martin, M.-J.; Orchard, S.; Magrane, M.; Agivetova, R.; Ahmad, S.; Alpi, E.; Bowler-Barnett, E.H.; Britto, R.; et al. UniProt: The universal protein knowledgebase in 2021. *Nucleic Acids Res.* **2021**, *49*, D480–D489, doi:10.1093/NAR/GKAA1100.
3. Vasaikar, S.V.; Deshmukh, A.P.; den Hollander, P.; Addanki, S.; Kuburich, N.A.; Kudaravalli, S.; Joseph, R.; Chang, J.T.; Soundararajan, R.; Mani, S.A. EMTome: A resource for pan-cancer analysis of epithelial-mesenchymal transition genes and signatures. *Br. J. Cancer* **2020**, *124*, 259–269, doi:10.1038/s41416-020-01178-9.
4. Ochoa, D.; Hercules, A.; Carmona, M.; Suveges, D.; Gonzalez-Uriarte, A.; Malangone, C.; Miranda, A.; Fumis, L.; Carvalho-Silva, D.; Spitzer, M.; et al. Open Targets Platform: Supporting systematic drug-target identification and prioritisation. *Nucleic Acids Res.* **2021**, *49*, D1302–D1310, doi:10.1093/nar/gkaa1027.
